# Progression of regional grey matter atrophy in multiple sclerosis

**DOI:** 10.1093/brain/awy088

**Published:** 2018-05-08

**Authors:** Arman Eshaghi, Razvan V Marinescu, Alexandra L Young, Nicholas C Firth, Ferran Prados, M Jorge Cardoso, Carmen Tur, Floriana De Angelis, Niamh Cawley, Wallace J Brownlee, Nicola De Stefano, M Laura Stromillo, Marco Battaglini, Serena Ruggieri, Claudio Gasperini, Massimo Filippi, Maria A Rocca, Alex Rovira, Jaume Sastre-Garriga, Jeroen J G Geurts, Hugo Vrenken, Viktor Wottschel, Cyra E Leurs, Bernard Uitdehaag, Lukas Pirpamer, Christian Enzinger, Sebastien Ourselin, Claudia A Gandini Wheeler-Kingshott, Declan Chard, Alan J Thompson, Frederik Barkhof, Daniel C Alexander, Olga Ciccarelli

**Affiliations:** 1 Queen Square Multiple Sclerosis Centre, UCL Institute of Neurology, Faculty of Brain Sciences, University College London, London, UK; 2 Centre for Medical Image Computing (CMIC), Department of Computer Science, University College London, UK; 3 Translational Imaging Group, Centre for Medical Image Computing (CMIC), Department of Medical Physics and Bioengineering, University College London, London, UK; 4 National Institute for Health Research (NIHR), University College London Hospitals (UCLH) Biomedical Research Centre (BRC), London, UK; 5 Department of Medicine, Surgery and Neuroscience, University of Siena, Siena, Italy; 6 Department of Neurosciences, S Camillo Forlanini Hospital, Rome, Italy; 7 Department of Neurology and Psychiatry, University of Rome Sapienza, Rome, Italy; 8 Neuroimaging Research Unit, Institute of Experimental Neurology, Division of Neuroscience, San Raffaele Scientific Institute, Vita-Salute San Raffaele University, Milan, Italy; 9 MR Unit and Section of Neuroradiology, Department of Radiology, Hospital Universitari Vall d'Hebron, Universitat Autònoma de Barcelona, Barcelona, Spain; 10 Department of Neurology/Neuroimmunology, Multiple Sclerosis Centre of Catalonia (CEMCAT), Hospital Universitari Vall d'Hebron, Universitat Autònoma de Barcelona, Barcelona, Spain; 11 Department of Anatomy and Neurosciences, VUmc MS Center, Neuroscience Amsterdam, VU University Medical Center, Amsterdam, The Netherlands; 12 Department of Radiology and Nuclear Medicine, MS Center Amsterdam, Amsterdam, The Netherlands; 13 Department of Neurology, MS Center Amsterdam, VU University Medical Center, Amsterdam, The Netherlands; 14 Department of Neurology, Medical University of Graz, Graz, Austria; 15 Division of Neuroradiology, Department of Radiology, Medical University of Graz, Graz, Austria; 16 Department of Brain and Behavioral Sciences, University of Pavia, Pavia, Italy; 17 Brain MRI 3T Research Centre, IRCCS Mondino Foundation, Pavia, Italy

**Keywords:** multiple sclerosis, atrophy, grey matter, probabilistic modelling, disability accumulation

## Abstract

See Stankoff and Louapre (doi:10.1093/brain/awy114) for a scientific commentary on this article.

Grey matter atrophy is present from the earliest stages of multiple sclerosis, but its temporal ordering is poorly understood. We aimed to determine the sequence in which grey matter regions become atrophic in multiple sclerosis and its association with disability accumulation. In this longitudinal study, we included 1417 subjects: 253 with clinically isolated syndrome, 708 with relapsing-remitting multiple sclerosis, 128 with secondary-progressive multiple sclerosis, 125 with primary-progressive multiple sclerosis, and 203 healthy control subjects from seven European centres. Subjects underwent repeated MRI (total number of scans 3604); the mean follow-up for patients was 2.41 years (standard deviation = 1.97). Disability was scored using the Expanded Disability Status Scale. We calculated the volume of brain grey matter regions and brainstem using an unbiased within-subject template and used an established data-driven event-based model to determine the sequence of occurrence of atrophy and its uncertainty. We assigned each subject to a specific event-based model stage, based on the number of their atrophic regions. Linear mixed-effects models were used to explore associations between the rate of increase in event-based model stages, and T_2_ lesion load, disease-modifying treatments, comorbidity, disease duration and disability accumulation. The first regions to become atrophic in patients with clinically isolated syndrome and relapse-onset multiple sclerosis were the posterior cingulate cortex and precuneus, followed by the middle cingulate cortex, brainstem and thalamus. A similar sequence of atrophy was detected in primary-progressive multiple sclerosis with the involvement of the thalamus, cuneus, precuneus, and pallidum, followed by the brainstem and posterior cingulate cortex. The cerebellum, caudate and putamen showed early atrophy in relapse-onset multiple sclerosis and late atrophy in primary-progressive multiple sclerosis. Patients with secondary-progressive multiple sclerosis showed the highest event-based model stage (the highest number of atrophic regions, *P < *0.001) at the study entry. All multiple sclerosis phenotypes, but clinically isolated syndrome, showed a faster rate of increase in the event-based model stage than healthy controls. T_2_ lesion load and disease duration in all patients were associated with increased event-based model stage, but no effects of disease-modifying treatments and comorbidity on event-based model stage were observed. The annualized rate of event-based model stage was associated with the disability accumulation in relapsing-remitting multiple sclerosis, independent of disease duration (*P < *0.0001). The data-driven staging of atrophy progression in a large multiple sclerosis sample demonstrates that grey matter atrophy spreads to involve more regions over time. The sequence in which regions become atrophic is reasonably consistent across multiple sclerosis phenotypes. The spread of atrophy was associated with disease duration and with disability accumulation over time in relapsing-remitting multiple sclerosis.

## Teaser

Grey matter atrophy in multiple sclerosis affects certain areas preferentially. Eshaghi *et al.* use a data-driven computational model to predict the order in which regions atrophy, and use this sequence to stage patients. Atrophy begins in deep grey matter nuclei and posterior cortical regions, before spreading to other cortical areas.

## Introduction

Multiple sclerosis is an inflammatory demyelinating disease of the CNS with a prominent neurodegenerative component. Brain atrophy, as assessed by MRI, develops at a faster rate in patients with multiple sclerosis than healthy control subjects. Whole brain atrophy is the result of grey matter, and, to a lesser extent, white matter atrophy (Fisher *et al.*, 2008), and is related to long-term disability in multiple sclerosis (Fisniku *et al.*, 2008; Filippi *et al.*, 2013). Histology studies have demonstrated that imaging-derived grey matter atrophy reflects neurodegeneration (Filippi *et al.*, 2012).

Grey matter atrophy is not uniform across the brain in multiple sclerosis, and some regions are more susceptible to atrophy than others (Steenwijk *et al.*, 2016; Preziosa *et al.*, 2017). The limbic system, temporal cortex and deep grey matter show rapid atrophy in patients with relapse-onset multiple sclerosis (Audoin *et al.*, 2010), while the cingulate cortex shows early atrophy in primary-progressive multiple sclerosis (Eshaghi *et al.*, 2014). In our previous study using the same large cohort of multiple sclerosis patients (Eshaghi *et al.*, 2018), we found that the deep grey matter showed the fastest annual rate of tissue loss in relapsing-remitting multiple sclerosis and progressive multiple sclerosis, and that in the cortex the rate of atrophy accelerated in the temporal regions in secondary-progressive multiple sclerosis. However, it is unknown whether there is a consistent and identifiable order in which atrophy progresses affecting different areas over time. A key question is whether there is an association between the sequential development of atrophy and disability accumulation.

One approach to investigate the sequence of atrophy progression is to use a probabilistic data-driven method, such as an event-based model, which, as the name implies, identifies the sequence of events at which a biomarker becomes abnormal, using cross-sectional or longitudinal observations (Fonteijn *et al.*, 2012; Young *et al.*, 2014). The event-based model is an established method. It has given new insights into the progression of Alzheimer’s disease in which the hippocampal atrophy is seen before the whole brain atrophy. Similarly, in Huntington’s disease, the event-based model has detected the earlier atrophy in the basal ganglia than other regions (Fonteijn *et al.*, 2012; Young *et al.*, 2014).

In this study, we have introduced a novel validation technique for the event-based model and then used it to investigate the progression of brain atrophy as a sequence of ‘events’ at which grey matter regions become atrophic in all phenotypes of multiple sclerosis. To define when the volume of a region ceases to be normal and becomes atrophic, the event-based model does not rely on *a priori* thresholds but calculates the probability of atrophy based on data-derived model distributions of normal and atrophic regional volumes. Moreover, the event-based model constructs a subject staging system: it assigns each subject to a stage that reflects how far through the sequence of regions that subject shows lower than normal volumes—the higher the stage, the higher the number of atrophic areas.

In this study, we built on the evidence that neurodegeneration in multiple sclerosis does not affect all the grey matter regions equally (Haider *et al.*, 2016; Eshaghi *et al.*, 2018) and those brain regions become atrophic in a non-random manner (Rocca *et al.*, 2010). We hypothesized that: (i) there is a sequence in which grey matter regions become atrophic; (ii) this sequence differs between relapse- and progressive-onset multiple sclerosis phenotypes; and (iii) the event-based model stage increases with disease duration and disability worsening.

## Materials and methods

### Participants

This was a retrospective study of 1424 participants, studied between 1996 and 2016 in seven European centres, which were part of the Magnetic Resonance in Multiple Sclerosis (MAGNIMS) Collaboration (www.magnims.eu). The same participants were previously used to investigate the spatiotemporal pattern of grey matter atrophy in multiple sclerosis (Eshaghi *et al.*, 2018). Subjects comprised healthy controls, patients with the clinically isolated syndrome, relapsing-remitting multiple sclerosis, secondary-progressive multiple sclerosis and primary-progressive multiple sclerosis. Eligibility criteria included: (i) a diagnosis of clinically isolated syndrome or multiple sclerosis according to the 2010 McDonald Criteria (Polman *et al.*, 2011); (ii) healthy controls without history of neurological or psychiatric disorders; (iii) the presence of at least two sequential MRI scans, acquired with an identical protocol, including T_1_-weighted MRI and T_2_-weighted/fluid attenuated inversion recovery (FLAIR) sequences; and (iv) a minimum interval of 6 months between scans. We requested that the Expanded Disability Status Scale (EDSS) score at clinical follow-ups on the eligible patients be made available (Kurtzke, 1983).

An additional group of age-matched healthy controls (*n* = 29) was also obtained from the Parkinson’s Progression Markers Initiative (PPMI) database (www.ppmi-info.org/data) to match healthy controls’ age to that of patients.

Magnetic resonance scans were acquired under written consent obtained from each participant independently in each centre. The final protocol for this study was reviewed and approved by the European MAGNIMS collaboration for the analysis of pseudo-anonymized scans.

### MRI data and analysis

We collected 3D T_1_-weighted scans, in addition to T_2_/FLAIR imaging, from all centres except one. Details of the 13 different MRI protocols are shown in Supplementary Table 1.

The aim of the image analysis was to extract the volume of brain regions according to the Desikan-Killiany-Tourville protocol (Klein and Tourville, 2012) [as explained in detail elsewhere (Eshaghi *et al.*, 2018)]. Briefly, the main steps were as follows: after an N4-bias field correction (part of ANTs software, version 4.1.9), which adjusted for the inhomogeneous intensity of the T_1_-weighted scans (Tustison *et al.*, 2010), we performed T_1_ lesion filling (Battaglini *et al.*, 2012) to improve the accuracy of the segmentation with the co-registered T_2_ lesion masks. We then created an unbiased, within-subject template, and linearly transformed all the subject-specific T_1_ scans to this symmetric space, using Freesurfer version 5.3 (Reuter *et al.*, 2010, 2012; Reuter and Fischl, 2011). In the symmetric space, we segmented the T_1_ scans in the grey matter, white matter and CSF using the Geodesic Information Flows software (part of NiftySeg, http://cmictig.cs.ucl.ac.uk/niftyweb/) (Cardoso *et al.*, 2015). Finally, we calculated regional volumes in the cortex and deep grey matter (the volumes of respective regions were averaged between the left and right hemisphere), the brainstem, white matter, cerebellum and lateral ventricles, according to the Desikan-Killiany-Tourville protocol (http://braincolor.mindboggle.info/index.html) (Klein and Tourville, 2012).

### The event-based model

We used the event-based model, as described previously (Fonteijn *et al.*, 2011, 2012; Young *et al.*, 2014), to estimate the most likely sequence in which selected regions become atrophic over time (see below for details on region selection). We also repeated the same analysis using all brain regions to test the dependence of our findings on the region selection.

The event-based model assumes that a population of patients represents the whole trajectory of disease progression (Fonteijn *et al.*, 2011) and reconciles cross-sectional or short-term longitudinal data into a picture of the entire disease course. We, therefore, created separate event-based models for (i) relapse-onset patients (the clinically isolated syndrome, relapsing-remitting, and secondary-progressive multiple sclerosis); (ii) progressive-onset (or primary-progressive) patients; and (iii) all clinical phenotypes together (to develop a unique staging system for the whole cohort). We used the sequence estimated by the latter event-based model to stage patients by assigning them the most probable stage along the sequence.

**Figure 1:**
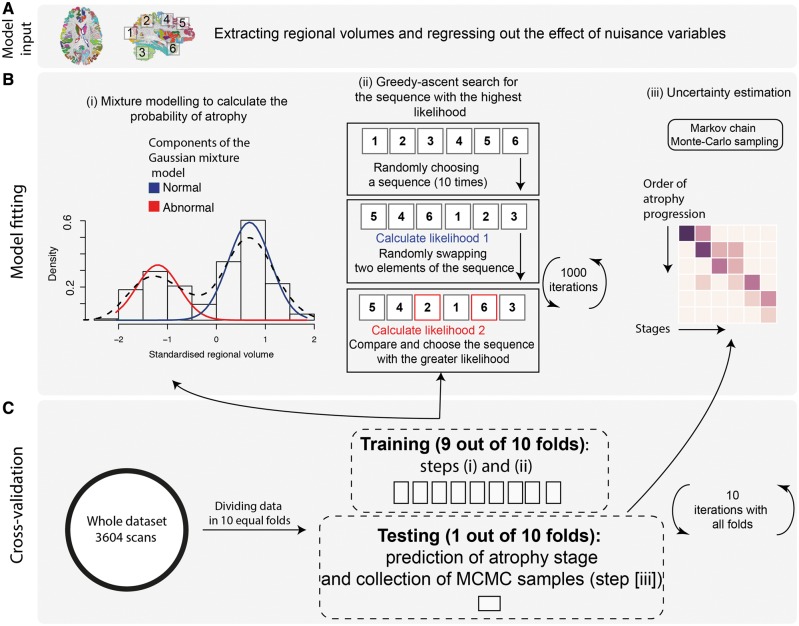
**The event-based model steps to estimate the most likely sequence of atrophy progression.** The three steps are: (**A**) adjusting for nuisance variables, and region selection; (**B**) calculating the best-fit probability distributions for normal and atrophic brain regions; searching for the most likely sequence; and (**C**) quantifying the uncertainty with cross-validation. [**B**(**i**)] The distribution of the volume in an example region in healthy controls and patients and the corresponding mixture model. (**ii**) The steps for greedy ascent search. (**iii**) A matrix showing a sequence of atrophy progression on the *y*-axis, and the position in the sequence of each region ranging from 1 to the total number of regions on the *x*-axis. The intensity of each matrix entry corresponds to the proportion of Markov Chain Monte Carlo samples of the posterior distribution where a certain region of *y*-axis appears at the respective stage of *x*-axis.

The main steps of the event-based model include (Fig. 1): (i) model input, which consists of the adjustment of regional volumes for effects of nuisance variables and selection of regions; (ii) model fitting; and (iii) a cross-validation. For the last step, we used a novel cross-validation method, used here within the event-based model for the first time, while steps (i) and (ii) have not changed since the original event-based model implementations (Fonteijn *et al.*, 2011; Young *et al.*, 2014). Model input used all multiple sclerosis patients. Model fitting and cross-validation were repeated three times using (i) relapse-onset and the clinically isolated patients together; (ii) primary-progressive multiple sclerosis; and (iii) the whole cohort of patients.

#### Model input

We adjusted the regional volumes for the total intracranial volume, age at study entry, gender, scanner magnetic field and MRI protocol. Since some centres provided data from more than one imaging protocol (Supplementary Table 1), we adjusted for imaging protocol and magnetic field (instead of ‘centre’). We constructed a regression model for each region separately, entering the volume as the dependent variable and the remaining variables as predictors. We extracted the amount of each regional volume that remained unexplained in the regression (residual of the fit). Subsequently, we selected the regions whose adjusted volumes at the study entry showed a significant difference between all multiple sclerosis patients and healthy controls, with a Bonferroni corrected *P < *0.01 (uncorrected *P* < 0.0001). We used these regions in the subsequent analyses. We then repeated the analysis using all the segmented regions of the Desikan-Killiany-Tourville atlas for the following reasons: (i) to test whether the sequence in which brain regions become atrophic was not influenced by restricting the analysis only to the regions that showed a lower volume in patients than controls; and (ii) to detect potential subtle early changes that might not have survived multiple-comparison correction.

#### Model fitting

The event-based model considers an ‘event’ to have occurred when a biomarker, here regional volume, has abnormal value (‘atrophy’) in comparison with the expected values measured in healthy controls. The model then estimates the sequence *S = S*(*1*), *S*(*2*), *…*, *S*(*l*) in which regions become atrophic, where *S*(*1*) is the first region, and *S*(*l*) is the last to become atrophic. The model assumes that all patients go through the same sequence as they progress. The estimation procedure first fits a mixture of two Gaussians to regional volumes, with one of the components fixed to be identical to the healthy distribution; the other component provides the model for the ‘abnormal’ distribution. This provides probabilistic models for normal and abnormal volumes from which we can calculate the likelihood of atrophy $P\left(x_{ij}\text{|}E_{i}\right)$ for the region *i* of the scan *j*, i.e. the probability density function (PDF) estimated at $x_{ij}$ from the abnormal component of the mixture model. The likelihood that region *i* has no atrophy or $P\left(x_{ij}\text{|}\neg E_{i}\right)$, is the PDF of the normal component of the mixture-model estimated at $x_{ij}$Fig. 1B(i)].

To search for the most likely sequence, we used a greedy ascent search (Fonteijn *et al.*, 2012; Young *et al.*, 2014) that started at 10 different random sequences and was iterated by randomly flipping sequences for 1000 times. The final sequence was selected when 10 different initial sequences converged to a similar likelihood after 1000 iterations. Within each iteration new (flipped) sequences [Fig. 1B(ii)] were accepted only if they increased the likelihood, which is defined as:

(1) $$P\left(X\text{|}S\right)=\overset{J}{\underset{j=1}{\prod }}\left[\overset{l}{\underset{k=0}{\sum }}(P\left(k\right)\overset{k}{\underset{i=1}{\prod }}P\left(x_{ij}\text{|}E_{i}\right)\;\overset{l}{\underset{i=k+1}{\prod }}P(x_{ij}|\neg E_{i})\;)\;\right]$$

where *X* is the data matrix, *S* is the sequence of atrophy events, *J* is the number of scans, *l* is the number of regions, and *P*(*k*) is the prior probability of being at stage *k*, which means *E_1_*, *…*, *E_k_* have occurred, and *E_k+1_*, …, *E_l_* have not occurred. We used a uniform distribution for prior probabilities, which assumes equal prior-probability for all possible stages; all sequences are equally likely *a priori*. The software and codes for the event-based model are freely available at https://github.com/ucl-mig/ebm.

#### Cross-validation of atrophy sequence

After estimating the most likely sequence, the uncertainty in the position of each region in the sequence was estimated using cross-validation and Markov Chain Monte Carlo. We divided the dataset (including baseline and follow-up visits) into 10 equally-sized folds (cross-validation folds) and repeated the sequence estimation 10 times. During each iteration, we used nine folds to fit the mixture-models (as explained above) and estimated the most-likely sequence. We kept one fold out as the test fold to assign the event-based model stages (explained below). Within each iteration, we used Markov Chain Monte Carlo to sample from the posterior distribution on the sequence given the nine-fold training data (Fonteijn *et al.*, 2012; Young *et al.*, 2014). We then aggregated Markov Chain Monte Carlo samples from the 10 iterations of cross-validation (10 000 samples from each fold) to calculate uncertainty across cross-validation folds. Finally, we used these 100 000 sampled sequences to plot the positional variance diagram (as in Fonteijn *et al.*, 2012; Young *et al.*, 2014), which shows on the *y*-axis the sequence with the highest likelihood, and the *x*-axis enumerates the number of sequence positions (or the event-based model stages). The intensities of the matrix entries correspond to the proportion of Markov Chain Monte Carlo samples in which the corresponding region (*y*-axis) appears at the respective stage (*x*-axis). Therefore, if there were no uncertainty, i.e. all Markov Chain Monte Carlo samples in all folds find the same sequence, the matrix would be black on the diagonal and white everywhere else; non-white off-diagonal and non-black diagonal elements indicate uncertainty in the position of the corresponding region in the sequence.

### Staging individual subjects and associations with white matter lesion load, disease duration and disability

We used the most likely sequence of atrophy progression from the whole patient cohort-based event-based model to obtain the event-based model stage for each scan *j*, which is the stage *k* that maximizes $\overset{k}{\underset{i=1}{\prod }}P(x_{ij}|E_{i})\overset{l}{\underset{i=k+1}{\prod }}P(x_{ij}|\neg E_{i})$. This assigned each subject an event-based model stage between 1 and the number of regions, *l*, at each visit (Fig. 1).

We used a nested linear mixed-effects model to investigate the association between the event-based model stage (dependent variable) and T_2_ lesion load (independent variable), in which time was nested in subject as the random-effect (to adjust for repeated measures). Similarly, we used a nested mixed-effects regression model to explore the association between the event-based model stage (dependent variable) and disease duration (independent variable), in which disease duration was nested in subject as the random effect.

For those clinical phenotypes that showed a significant change in the event-based model stage over time (relapsing-remitting, secondary-progressive and primary-progressive multiple sclerosis), we investigated whether longitudinal EDSS changes could be predicted by event-based model changes independent of disease duration. We divided the changes in the EDSS and event-based model by the number of years from the study entry and performed a linear regression analysis where the annualized EDSS change was the outcome variable. Annualized event-based model stage change and disease duration at the study entry were the predictor variables. Since both the event-based model stage and EDSS are ordinal variables, we used ordinal regression analyses to confirm the results of the linear regressions but presented the results of linear models (as they did not materially differ).

#### Confounding effects of disease-modifying treatments and comorbidities

To test whether disease-modifying treatments could affect the event-based model stages at baseline and over time, we used similar mixed-effects models (as above) in which the event-based model stage was the outcome variable; time, disease-modifying treatment (as a categorical variable), and their interaction were the fixed-effect variables. Random effects were the same as explained above. We performed additional analyses to assess the effects of comorbidities on the event-based model stages, which are reported in the Supplementary material.

## Results

### Subject characteristics

Imaging data from 1424 subjects were analysed; three subjects’ scans were excluded because of motion artefacts and four because of poor registration due to missing imaging header information. Therefore, data from 1417 subjects were included in the final modelling: 1214 patients (253 clinically isolated syndrome, 708 relapsing-remitting, 128 secondary-progressive, and 125 primary-progressive multiple sclerosis), and 203 healthy controls. The average [± standard deviation (SD)] length of follow-up for patients was 2.43 years (±1.97) and for healthy controls was 1.83 years (±1.77). In total, we analysed 3604 T_1_-weighted scans [mean number of scans per patient was 2.54 (SD = 1.04)] (Table 1).

### Sequence of atrophy progression

**Figure 2:**
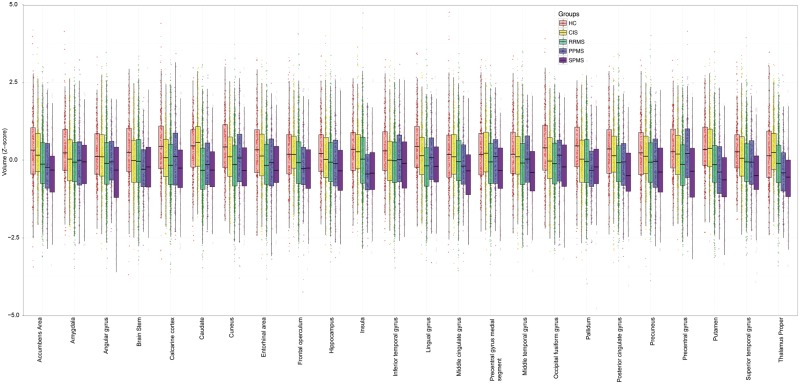
**Comparisons of regional volumes between groups.** Box plots at *y*-axis show *z*-scores of the corresponding region shown at *x*-axis. Lower and upper hinges of each boxplot correspond to 25th and 75th percentiles of data. We selected 24 regions that showed significant difference (*P* < 0.01 corrected) between all patients with multiple sclerosis and healthy controls at baseline visit. CIS = clinically isolated syndrome; HC = healthy control; PPMS = primary progressive multiple sclerosis; RRMS = relapsing-remitting multiple sclerosis; SPMS = secondary progressive multiple sclerosis.

At baseline, 24 regions showed a smaller volume in multiple sclerosis than healthy controls (Bonferroni corrected *P < *0.01). They included the deep grey matter regions and the posterior cortices (including the precuneus and the posterior cingulate cortex), several regions in the temporal lobe, the precentral cortex, and the brainstem (see Fig. 2 for the full list).

**Figure 3:**
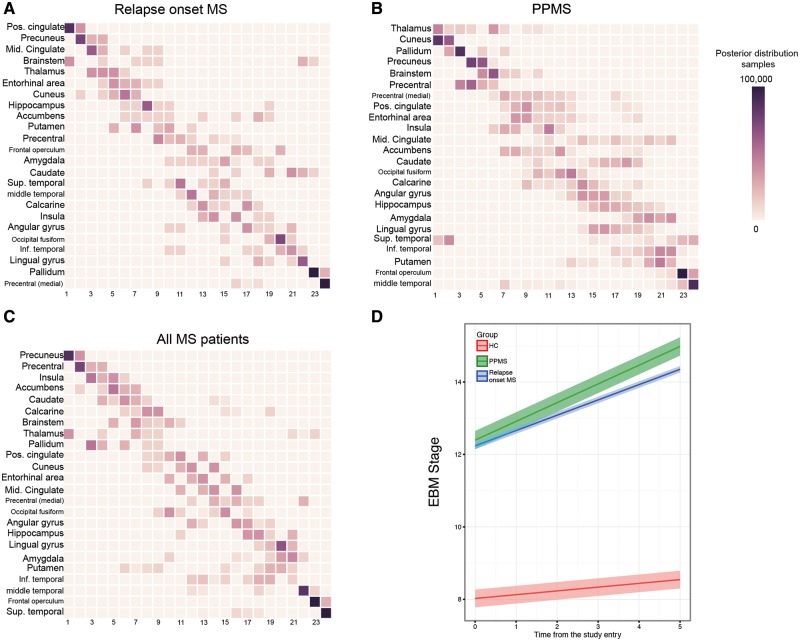
**Sequences of atrophy progression and patient staging.** The positional variance diagrams for (**A**) relapse-onset multiple sclerosis, (**B**) primary-progressive multiple sclerosis (PPMS) and (**C**) merged cohort of patients, show the most likely sequences of atrophy and their associated uncertainty. In **A**–**C**, the *y*-axis shows the most likely sequence of atrophy progression, and the *x*-axis shows the sequence position ranging from one to the total number of regions. The intensity of each rectangle corresponds to the proportion of Markov Chain Monte Carlo samples of the posterior distribution where a certain region of *y*-axis appears at the respective stage of the *x*-axis. (**D**) The evolution of the event-based model (EBM) stage (or atrophy progression staging) over time in clinically isolated syndrome and relapse-onset multiple sclerosis together and primary-progressive multiple sclerosis. Each line in **D** is the prediction of the mixed-effects model whose ribbon shows standard error of the prediction. MS = multiple sclerosis.

When we estimated the sequence in which these 24 regions become atrophic in patients with relapse-onset multiple sclerosis (i.e. relapsing-remitting and secondary-progressive) and the clinically isolated syndrome, the first regions were the posterior cingulate cortex and precuneus, followed by the middle cingulate cortex, brainstem, and thalamus (Fig. 3A and D); the last regions to become atrophic were the pallidum and medial precentral gyrus.

In patients with primary-progressive multiple sclerosis, among the 24 selected regions, the first ones to show atrophy were the thalamus, cuneus and precuneus, and pallidum, followed by the brainstem, precentral gyrus, and posterior cingulate cortex (Fig. 3B and D); the last regions to become atrophic were the frontal operculum and middle temporal gyrus.

When the event-based model was used to estimate the sequence of atrophy progression of the selected 24 regions in all patients together, additional regions were detected as showing early atrophy, such as the insula, accumbens and caudate (Fig. 3C). The likelihood of the 10 randomly chosen sequences (log-likelihood range: −149 000 to −117 000) converged to a similar range (log-likelihood range: −1 000 000 to −99 000) after 1000 iterations (Supplementary Fig. 1). For other event-based models, the likelihoods converged to a similar range (results are not shown).

**Figure 4:**
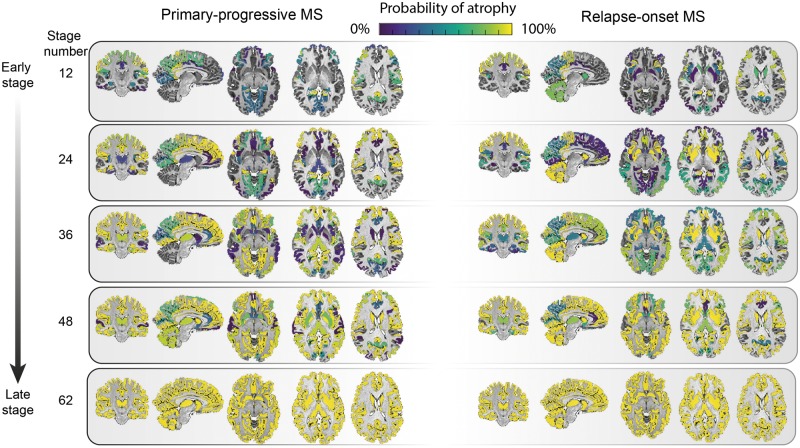
**Regional atrophy and its sequence of progression in all grey matter regions plus brainstem in relapse-onset disease and primary progressive multiple sclerosis.** The probability of atrophy in each region was calculated from the positional variance diagrams and colour coded, so that brighter colour corresponded to a higher probability of seeing atrophy in the corresponding event-based model stage. MS = multiple sclerosis.

When all the remaining regions were included additional regions were identified. In primary-progressive multiple sclerosis, they were the transverse temporal gyrus, cerebral white matter, post-central gyrus and middle frontal gyrus (Fig. 4 and Supplementary Figs 2 and 3). In the relapse-onset group, these regions were the superior frontal gyrus, inferior frontal gyrus, and middle frontal gyrus.

When we qualitatively compared the clinically isolated syndrome and relapse-onset multiple sclerosis patients with primary-progressive multiple sclerosis, across all regions, the cerebellum, caudate and putamen showed a differential pattern of atrophy, with early atrophy in patients with relapse-onset disease and late atrophy in primary progressive multiple sclerosis (Fig. 4).

### Event-based model staging of individual subjects

Patients with clinically isolated syndrome and relapse-onset multiple sclerosis and primary-progressive multiple sclerosis had significantly higher event-based model stages at baseline than healthy controls [average intercept (± standard error (SE)] of the event-based model stage for healthy control subjects = 8.02 (±0.59), relapse-onset = 12.39 (±0.66), primary-progressive multiple sclerosis = 12.22 (±0.35), *P < *0.05]; when looking at each clinical phenotype, patients with secondary-progressive multiple sclerosis had the highest event-based model stage at the study entry [14.73 (±0.93), all *P*-values < 0.001], followed by relapsing-remitting [12.60 (±0.67)], primary-progressive multiple sclerosis [12.22 (±0.35)], clinically isolated syndrome [8.12 (±0.76)], and healthy controls [8.02 (±0.59)]. The annual rate of change (or slope) in the event-based model stage over time was significant (null-hypothesis = zero slope) for secondary-progressive multiple sclerosis [average slope (±SE) = 1.02 (±0.41)], primary-progressive [0.52 (±0.34)], and relapsing-remitting multiple sclerosis [0.37 (±0.26)], but not for clinically isolated syndrome [0.19 (±0.33)] and healthy controls [0.10 (±0.24)]. The rate of change, although nominally higher in secondary progressive multiple sclerosis, was not significantly different between clinical phenotypes.

### Association of the event-based model stages with white matter T_2_ lesion load, disease duration and disability accumulation

At baseline, the highest T_2_ lesion load was observed in secondary-progressive multiple sclerosis, followed by primary-progressive multiple sclerosis (Table 1). There was a significant association between the event-based model stage and white matter lesion load (standardized *β* = 0.11, *P < *0.001) in all patients, which means for every standard deviation (15.31 ml) increase in the lesion load there was a 0.11 SD (1.06 unit) increase in the event-based model stage. However, there was no association between the rate of change in the event-based model stage over time and the rate of increase in lesion load.

**Table 1:** Baseline characteristics of participants

Group	Healthy control	Clinically isolated syndrome	Relapse-onset multiple sclerosis^a^	Primary- progressive multiple sclerosis
*n* (females, *n*)	203 (112)	253 (171)	836 (548)	125 (55)
Age (±SD)	38.7 ± 10.5	33 ± 8	39.7 ± 9.8	48.5 ± 10.1
Disease duration (±SD)	–	0.4 ± 1.4	8.06 ± 8.03	6.8 ± 5.9
Median EDSS (range)	–	1 (0–4.5)	2 (0–9)	5 (2–8)
Per cent (*n*) of patients receiving disease-modifying treatments	–	20 (52)	47 (397)	6 (8)
Baseline median white matter T_2_ lesion load (ml) (1st–3rd quartile)	–	2.97 (1.01–5.04)	5.04 (2.05–11.79)^b^	9.38 (2.69–22.02)

^a^Relapse-onset group includes both the relapsing-remitting and secondary-progressive patients.

^b^Baseline median T_2_ lesion load were the following: for relapsing-remitting = 5.05 (2.05–11.79) and secondary-progressive multiple sclerosis = 11.04 (3.18–23.14).

There was a significant association between the rate of increase in the event-based model stages and disease duration in all patients with multiple sclerosis (*β* = 0.21, SE = 0.03, *P < *0.001) using all available time points. This means that for every increase of one event-based model stage, disease duration increased by 4.76 years.

At the baseline visit, there was no significant association between the event-based model stage and the EDSS in any clinical phenotype. Over time there was a significant increase in the EDSS in both relapse-onset multiple sclerosis/clinically isolated syndrome and primary-progressive multiple sclerosis patients (an increase of 0.07 and 0.2 per year, respectively, *P < *0.01). There was a significant association (independent of disease duration) between the annualized event-based model stage and the annualized EDSS changes in relapsing-remitting multiple sclerosis (beta = 0.03, *P < *0.0001), but not in secondary-progressive and primary-progressive multiple sclerosis. This means that assuming a linear relationship between the EDSS and the event-based model stage, for every unit increase in the annual rate of the event-based model stage there is 0.03 increase in the annual rate of EDSS worsening.

### Disease-modifying treatments and comorbidity did not affect the event-based model stages

Information on whether or not a patient was receiving a disease-modifying treatment was available for 98% of patients (*n = *1179) at baseline. Of these, 38% (*n = *457) were receiving a disease-modifying therapy; 47% of patients with relapse-onset multiple sclerosis, 20% of patients with clinically isolated syndrome and 6% of patients with primary-progressive multiple sclerosis were on treatment (Table 1). Information on the type of disease-modifying treatment was available for 56% of these patients (*n = *255), of whom 86% (*n = *220) were receiving either interferons or glatiramer acetate, and the remaining 14% (*n = *35) of patients were on other treatments, including natalizumab, fingolimod, mitoxantrone, and teriflunomide. Linear mixed-effects models showed that at baseline (estimated average ± SE) the event-based model stage was not significantly different (*P = *0.21) between patients who were on disease-modifying treatments (12.63 ± 0.32) compared to those who were not (11.98 ± 0.52). The same model showed that the annual rate of change (estimated range ± SE) in the event-based model stage was not significantly different (*P = *0.45) between patients who were on disease-modifying treatments (0.53 ± 0.17) and those who were not (0.39 ± 0.10).

Similarly, we found negative results on whether the event-based model stage and its rate of change over time differed between patients with and without comorbidity (Supplementary material).

## Discussion

In this study, we used a data-driven method to determine the most likely sequence in which brain regions become atrophic in multiple sclerosis. This sequence is consistent in key regions across multiple sclerosis phenotypes: the posterior cingulate cortex, precuneus, and thalamus were among the earliest regions to become atrophic in both relapse-onset phenotypes and primary-progressive multiple sclerosis. The event-based model staging system was applied to individual patients, and the rate of increase in the event-based model stage was associated with the disease duration in all multiple sclerosis phenotypes and with the EDSS in patients with relapsing-remitting multiple sclerosis independent of the disease duration. These results provide insights into the mechanisms of disease worsening in multiple sclerosis.

The order of atrophy progression in the event-based model for most regions was similar between primary-progressive multiple sclerosis and the clinically isolated syndrome/relapse-onset multiple sclerosis. This may support the evidence from histological studies that the pathological processes are regionally consistent between early relapsing-remitting and progressive multiple sclerosis (Mahad *et al.*, 2015). Our results showed that areas with an early atrophy were the posterior cingulate cortex, precuneus, thalamus and brainstem in both groups, thereby extending the results of previous studies, which have limited their investigation to specific multiple sclerosis subtypes (Gilmore *et al.*, 2009; Audoin *et al.*, 2010; Calabrese *et al.*, 2015*b*; Steenwijk *et al.*, 2016). When all patients were included together, the insula, accumbens and caudate were predicted as becoming atrophic early on.

The cingulate cortex and insula have extensive connections with other regions. Possible factors for their early atrophy, therefore, can include disconnection secondary to white matter lesions, inflammation, and more specifically meningeal inflammation. We, therefore, calculated white matter T_2_ lesion volumes and showed that there was an association between increasing lesion load at baseline and the event-based model stages. Since assessing meningeal inflammation is challenging *in vivo* we can just speculate that structures in cortical invaginations can be exposed to meningeal inflammation, cortical demyelination, and neurodegeneration (Gilmore *et al.*, 2009; Howell *et al.*, 2011; Haider *et al.*, 2016). The cingulate cortex and precuneus are part of a network of active regions during rest (the default mode network) (Raichle, 2015). These regions are interconnected with other areas, have the highest energy consumption in the brain, and are affected by multiple sclerosis and other neurodegenerative disorders (Bonavita *et al.*, 2011; Raichle, 2015). In multiple sclerosis, neurons with demyelinated axons consume more energy to adapt to demyelination, which creates a micro-environment similar to that of hypoxia (‘virtual hypoxia’) (Trapp and Stys, 2009). Neurons that survive in a state of persistent virtual hypoxia are more vulnerable to degeneration (Zhang and Raichle, 2010), and this may explain the higher vulnerability of the cingulate and precuneus cortex to atrophy.

Other regions that showed early atrophy were the thalamus and the brainstem in both relapse-onset multiple sclerosis and primary-progressive multiple sclerosis. In our previous study, we found that the deep grey matter showed the fastest rate of atrophy over time, while brainstem had the highest atrophy (the lowest volume) at study entry, but its atrophy progressed at a slower rate than that occurring in other regions (Eshaghi *et al.*, 2018). This may suggest that during early stages of multiple sclerosis, the rate of atrophy in the brainstem is higher than later stages, while the rate of atrophy in the thalamus remains high throughout the disease course. The brainstem is in close contact with the spinal cord, whose atrophy is seen from early stages of multiple sclerosis independent of the cortex or deep grey matter (Biberacher *et al.*, 2015; Ruggieri *et al.*, 2015).

Several mechanisms may underlie neurodegeneration in the deep grey matter, including mitochondrial failure, iron deposition, retrograde degeneration through white matter lesions, and meningeal inflammation (for structures closer to CSF) (Calabrese *et al.*, 2015*a*; Bodini *et al.*, 2016; Haider *et al.*, 2016; Pardini *et al.*, 2016). Network overload and collapse, similar to the cingulate and precuneus cortex, could also explain preferential atrophy of the deep grey matter in multiple sclerosis (Minagar *et al.*, 2013).

There were a few regions showing a differential pattern of atrophy between relapse- and progressive-onset phenotypes. The cerebellum, caudate and putamen were predicted to have early atrophy in relapse-onset disease and late atrophy in primary-progressive multiple sclerosis. In the cerebellum, this different behaviour can be explained by a more inflammatory phenotype of patients with relapse-onset multiple sclerosis. In patients with multiple sclerosis, more than any other brain region, demyelination is seen in the cerebellar grey matter, which is five times more than the white matter demyelination (Gilmore *et al.*, 2009). This may be a consequence of overlying meningeal inflammation in the deep folia, which accommodate a static inflammatory milieu (such as cytokines, and immunoglobulins) (Kutzelnigg *et al.*, 2007; Howell *et al.*, 2011). Therefore, in the cerebellum, overlying inflammation may play a role and amplify other pathological mechanisms, such as retrograde neurodegeneration secondary to white matter lesions (Kutzelnigg *et al.*, 2007; Gilmore *et al.*, 2009; Howell *et al.*, 2011). Thus, the cerebellum could be susceptible to inflammatory damage from the CSF. Previous studies have reported in relapse-onset multiple sclerosis, but not primary-progressive multiple sclerosis, tertiary lymphatic follicles in cortical invaginations, which may suggest a more inflammatory CSF milieu than primary-progressive multiple sclerosis (Kutzelnigg *et al.*, 2007; Choi *et al.*, 2012). This could explain earlier atrophy of the cerebellar grey matter in people with relapse onset disease, while in primary-progressive multiple sclerosis, neurodegeneration in a less inflammatory CSF milieu might cause a gradual progression of atrophy (Choi *et al.*, 2012; Mahad *et al.*, 2015). However, this is speculative, and it remains unclear whether meningeal inflammation has a causative effect on demyelination and neurodegeneration.

The caudate and putamen, which are histologically similar, constitute a structure that is known as the neostriatum. A previous histopathological study has shown that the greatest extent of demyelination and lesions in the deep grey matter can be seen in the caudate even in early multiple sclerosis, although the pattern was not different between multiple sclerosis phenotypes (Haider *et al.*, 2014). Moreover, the putamen receives significant inputs from the motor cortex and the caudate from the association cortices. Therefore, we could speculate that retrograde neurodegeneration secondary to a higher lesion load in relapse-onset disease (compared to primary-progressive multiple sclerosis) may perform as an additive factor on demyelination to explain the higher vulnerability of these structures.

We extended our analysis from regions that showed significant atrophy at baseline to the all segmented areas to test the dependence of our findings to region selection. Another reason was to explore early, but subtle changes in brain regions, which might have been missed by just looking at a snapshot at the study entry to choose specific areas whose adjusted volumes showed a significant difference between all multiple sclerosis patients and healthy controls, based on stringent multiple-comparison correction. For example, a brain region may show mild volume loss earlier than another part with a greater (but later) volume loss through the course of multiple sclerosis. Whole brain event-based model analysis predicted an early involvement of the posterior cortices (posterior cingulate and precuneus) along with the brainstem. New additional regions in the whole brain event-based model were also identified as showing atrophy at an early stage, including the superior, middle, and inferior frontal gyri in relapse-onset phenotypes, and the transverse temporal gyrus, white matter, and post-central gyrus in primary-progressive multiple sclerosis. These findings suggest that the changes in these structures may happen early, but with a lower intensity than other regions that were selected initially (24 areas).

This study was not designed to investigate the effects of disease-modifying drugs and comorbidities on the atrophy stages. However, it does study the sequence of regional atrophy in the presence of these confounders. There were no significant differences at baseline or during the follow-up in the event-based model stages of patients who were receiving disease-modifying treatments and those who were not, extending our previous results in the same group of patients which demonstrated, using a different imaging analysis method, that the rates of atrophy in neuroanatomical regions were not confounded by disease-modifying treatments (Eshaghi *et al.*, 2018). Most of the patients were receiving the injectable first-line therapies (interferon beta and glatiramer acetate), whose effects on atrophy rates are weak (Filippi *et al.*, 2004; Cohen *et al.*, 2010). Although the information on comorbidities was only available for about a third of patients, we found that patients with at least one comorbidity were older at baseline than those without. This is in line with the literature showing that comorbidities are prevalent in patients with multiple sclerosis and increase with age (Marrie and Horwitz, 2010; Geraldes *et al.*, 2017). There was no significant effect of comorbidity on event-based model stage at baseline or its rate of change during the follow-up. One reason that age influenced the frequency of comorbidities, but not the number of atrophic regions, was that we had regressed out the effects of age on the regional volumes. Therefore, we conclude that disease-modifying treatments and comorbidity did not significantly influence our findings.

The event-based model has a potential for clinical use as it does not rely on time and can be applied to individual (cross-sectional) brain scans. To have a unique staging system across all clinical phenotypes, we created an event-based model from the whole patient cohort. We showed that patients with secondary-progressive multiple sclerosis had the highest event-based model stage—or the highest number of atrophic regions—at the study entry. This, in line with previous studies, suggests that secondary-progressive multiple sclerosis has more advanced neurodegeneration across multiple sclerosis phenotypes (Ceccarelli *et al.*, 2008). When we performed the event-based model staging using follow-up scans of patients and healthy controls, we found a significant increase in event-based model stages in all multiple sclerosis phenotypes, but not in the clinically isolated syndrome or healthy controls (although the baseline event-based model stage was nominally higher in the clinically isolated syndrome than healthy controls). The clinical relevance of the event-based model was confirmed by a significant association between stages and EDSS in relapsing-remitting multiple sclerosis, after adjusting for disease duration. Therefore, the sequential pattern of atrophy may explain disease worsening in relapsing-remitting multiple sclerosis. We did not find the same association between the changes in event-based model stages and EDSS in other patient groups. However, patients with secondary-progressive multiple sclerosis had the highest event-based model stages at the study entry and the highest (nominal) rate of increase in the event-based model stage.

Although this is a retrospective and multi-centre study, we have adjusted for the effects of MRI protocol and scanner magnetic field, and, as reported before on this dataset (Eshaghi *et al.*, 2018), the effect of multiple sclerosis phenotypes on regional measures was higher than that from these variables. A possible limitation is that the event-based model assumes that all brain regions eventually become abnormal (all regions show atrophy at the last stage). Therefore, an implicit assumption is that patients with relapse-onset disease (the clinically isolated syndrome, relapsing-remitting, and secondary-progressive multiple sclerosis) represent the whole continuum of progression when analysed separately; future implementations of this model could remove this assumption. We used the EDSS as the clinical outcome, but both the EDSS and event-based model provide measures that are ordinal, and may not have a uniform interpretation. Therefore, the coefficients of associations should be interpreted relatively (e.g. to compare clinical groups) rather than absolutely.

We showed that the sequence of atrophy progression in relapse-onset disease and primary-progressive multiple sclerosis are similar in many key regions, while the cerebellum, caudate and putamen show an earlier atrophy in relapse-onset multiple sclerosis than primary-progressive multiple sclerosis, perhaps due to a more inflammatory milieu. The sequence of atrophy progression can be used to score patients during multiple sclerosis automatically.

## Funding

A.E. receives the McDonald Fellowship from Multiple Sclerosis International Federation (MSIF, http://www.msif.org) and ECTRIMS-MAGNIMS fellowship. This study has received funding from European Union's Horizon 2020 research and innovation programme under grant agreement Nos 634541 and 666992 (EuroPOND project). D.A has received funding for this study from EPSRC (M020533, M006093, J020990). This research was supported by the National Institute for Health Research (NIHR) University College London Hospitals (UCLH) Biomedical Research Centre (BRC). A.J.T., O.C. and F.B. acknowledge support from the UCL/UCLH National Institute of Health (NIHR) Biomedical Research Centre (BRC). PPMI—a public-private partnership—is funded by the Michael J. Fox Foundation for Parkinson’s Research and funding partners (see the full list at www.ppmi-info.org/fundingpartners).

## Conflicts of interest

C.T. received an ECTRIMS post-doctoral research fellowship in 2015. She has also received honoraria and support for travelling from Teva Pharmaceuticals Europe and Ismar Healthcare. F.P. has received a Guarantors of Brain Fellowship. N.De.S. has received honoraria from Biogen-Idec, Genzyme, Merck Serono, Novartis, Roche and Teva for consulting services, speaking and travel support. He serves on advisory boards for Merck Serono, Novartis, Biogen-Idec, Roche, and Genzyme; he has received research grant support from the Italian MS Society. C.E. received funding for traveling and speaker honoraria from Biogen Idec, Bayer Schering Pharma, Merck Serono, Novartis, Genzyme and Teva Pharmaceutical Industries Ltd./sanofi-Aventis; received research support from Merck Serono, Biogen Idec, and Teva Pharmaceutical Industries Ltd./sanofi-Aventis; and serves on scientific advisory boards for Bayer Schering Pharma, Biogen Idec, Merck Serono, Novartis, Genzyme, Roche, and Teva Pharmaceutical Industries Ltd./sanofi- Aventis. A.R. serves on scientific advisory boards for Biogen Idec, Novartis, Sanofi-Genzyme, and OLEA Medical has received speaker honoraria from Bayer, Sanofi-Genzyme, Bracco, Merck-Serono, Teva Pharmaceutical Industries Ltd, Novartis and Biogen Idec, and has research agreements with Siemens AG and Icometrix. M.A.R. has received speaker’s honoraria from Biogen Idec, Novartis, TEVA, Genzyme Sanofi-Aventis, Teva and Merk Serono and receives research support from the Italian Ministry of Health and Fondazione Italiana Sclerosi Multipla. M.F. is Editor-in-Chief of the Journal of Neurology; serves on scientific advisory board for Teva Pharmaceutical Industries; has received compensation for consulting services and/or speaking activities from Biogen Idec, Excemed, Novartis, and Teva Pharmaceutical Industries; and receives research support from Biogen Idec, Teva Pharmaceutical Industries, Novartis, Italian Ministry of Health, Fondazione Italiana Sclerosi Multipla, Cure PSP, Alzheimer's Drug Discovery Foundation (ADDF), the Jacques and Gloria Gossweiler Foundation (Switzerland), and ARiSLA (Fondazione Italiana di Ricerca per la SLA). C.E.L. reports a grant from Stichting multiple sclerosis research. H.V. has received research grants from Pfizer, Merck-Serono, Novartis and Teva, and speaker honoraria from Novartis and Merck Serono; all funds were paid directly to his institution. B.M.J.U. has received personal compensation for consulting from Biogen Idec, Genzyme, Merck Serono, Novartis, Roche en TEVA. C.A.G.W-K. receives research grants (PI and co-applicant) from ISRT, EPSRC, Wings for Life, UK MS Society, Horizon2020, Biogen and Novartis. C.G. has received fees as an invited speaker or travel expenses for attending meetings from Biogen, Merck-Serono, Teva, Sanofi, Novartis, Genzyme. S.R. has received speaking honoraria from Merck-Sereno and Teva and fees has travel expenses from Biogen. D.C. has received honoraria (paid to his employer) from Ismar Healthcare NV, Swiss multiple sclerosis Society, Excemed (previously Serono Symposia International Foundation), Merck, Bayer and Teva for faculty-led education work; Teva for advisory board work; meeting expenses from Merck, Teva, Novartis, the multiple sclerosis Trust and National multiple sclerosis Society; and has previously held stock in GlaxoSmithKline. D.A. has received funding for this work from EPSRC (M020533, M006093, J020990) as well as the *European Union’s Horizon 2020 research and innovation programme* under grant agreement Nos 634541 and 666992. In the past year, A.T. has received honoraria/support for travel from Eisai, Hoffmann-La Roche, and Excemed. He received support for travel from the International Progressive Multiple Sclerosis Alliance, as chair of their Scientific Steering Committee and the National Multiple Sclerosis Society (USA) as member of their Research Programs Advisory Committee. He receives an honorarium from SAGE Publishers as the Editor-in-Chief of MSJ. F.B. acts as a consultant to Biogen-Idec, Janssen Alzheimer Immunotherapy, Bayer-Schering, Merck-Serono, Roche, Novartis, Genzyme, and Sanofi-aventis. He has received sponsorship from EU-H2020, NWO, SMSR, EU-FP7, TEVA, Novartis, and Toshiba. He is on the editorial board of Radiology, Brain, Neuroradiology, MSJ, and Neurology. O.C. receives research grant support from the Multiple Sclerosis Society of Great Britain and Northern Ireland, the NIHR UCLH Biomedical Research Centre, the Rosetree Trust, the National multiple sclerosis Society and the NIHR-HTA; she is a consultant for Teva, Roche, Novartis, Biogen, Genzyme and GE. She is an Associate Editor for Neurology, for which she receives an honorarium. S.O. and L.P. have nothing to disclose.

## Supplementary material

Supplementary material is available at *Brain* online.

## Appendix 1

### MAGNIMS Steering Committee Members

Alex Rovira: MR Unit and Section of Neuroradiology, Department of Radiology, Multiple Sclerosis Centre of Catalonia (CEMCAT), Hospital Universitari Vall d'Hebron, Universitat Autònoma de Barcelona, Barcelona, Spain. Christian Enzinger: Department of Neurology, Medical University of Graz, Graz, Austria Frederik Barkhof: Queen Square Multiple Sclerosis Centre, UCL Institute of Neurology, University College London, London, UK. Olga Ciccarelli: Queen Square Multiple Sclerosis Centre, UCL Institute of Neurology, University College London, London, UK. Massimo Filippi: Neuroimaging Research Unit, Institute of Experimental Neurology, Division of Neuroscience, San Raffaele Scientific Institute, Vita-Salute San Raffaele University, Milan, Italy. Nicola De Stefano: Department of Medicine, Surgery and Neuroscience, University of Siena, Siena, Italy. Ludwig Kappos: Department of Neurology, University Hospital, Kantonsspital, Basel, Switzerland. Jette Frederiksen: The multiple sclerosis Clinic, Department of Neurology, University of Copenhagen, Glostrup Hospital, Denmark. Jaqueline Palace: Centre for Functional Magnetic Resonance Imaging of the Brain, University of Oxford, UK. Maria A Rocca: Neuroimaging Research Unit, Institute of Experimental Neurology, Division of Neuroscience, San Raffaele Scientific Institute, Vita-Salute San Raffaele University, Milan, Italy. Jaume Sastre-Garriga: Department of Neurology/Neuroimmunology, Multiple Sclerosis Centre of Catalonia (CEMCAT), Hospital Universitari Vall d'Hebron, Universitat Autònoma de Barcelona, Barcelona, Spain. Hugo Vrenken: Department of Radiology and Nuclear Medicine, multiple sclerosis Center Amsterdam, Amsterdam, The Netherlands. Tarek Yousry: NMR Research Unit, Institute of Neurology, University College London, London, UK. Claudio Gasperini: Department of Neurology and Psychiatry, University of Rome Sapienza, Rome, Italy.

## Supplementary Material

Supplementary Data

## Abbreviation

EDSS: Expanded Disability Status Scale
