# Supplementary material for: Progression of regional grey matter atrophy in multiple sclerosis
Source: Brain. 2018 May 8;141(6):1665–77. doi: 10.1093/brain/awy088 (PMC5995197; doi:10.1093/brain/awy088)
Supplement: Supplementary Data [file awy088_brain-2017-01792-file005.pdf]

Supplementary Methods

Effects of comorbidities on atrophy measures

We performed a subgroup analysis on the subset of patients with available comorbidity information. The comorbidity was defined as a categorical variable that showed the documented presence (or absence) of diabetes, hypertension, hyperlipidaemia, ischemic heart disease, stroke, other autoimmune diseases, psychiatric diagnosis (major depression disorder, bipolar disorder, alcohol abuse, and other psychiatric disorder), chronic lung disease, renal disorders, migraine, and smoking before the date of first scan (Ann Marrie et al. 2015 and Geraldes et al. 2017). We aimed to compare the effect of comorbidities on the rate of change in the event-based model stage. We used a linear mixed effects model in which the event-based model stage was the outcome variable. Time, comorbidity, and the interaction of comorbidity with time were the fixed-effect variables. Time was nested in the centre as the random-effect.

Supplementary Results

The data on comorbidities were available for 28% of patients (n=340) at baseline. Out of these patients, 183 patients (18 primary-progressive multiple sclerosis, 110 relapsing-remitting multiple sclerosis, 22 secondary-progressive multiple sclerosis, and 33 clinically isolated syndrome), had at least one comorbidity and the remaining 157 (23 primary progressive multiple sclerosis, 86 relapsing-remitting multiple sclerosis, 8 secondary-progressive multiple sclerosis, and 40 with the clinically isolated syndrome) had no comorbidity. At baseline, patients without comorbidity were significantly younger than those with comorbidity (37.27±9.79 vs 40.2 ±11.56, p=0.01). The number of atrophic brain regions (or the event-based model stage) did not differ between patients with and without comorbidity (11.03 (9.41) vs 9.08 (9.01), *p*=0.18). Similarly, the estimated annual rate of change (±standard error) in the event-based model stage did not differ between the two groups (0.575 (±0.22) vs 0.76 (±0.32), p=0.55, adjusted for age).

Supplementary Table 1. MRI protocols for each participating centre.

	T1-weighted MRI							Sequence for lesion delineation					
	**Magnetic field**	**Vendor**	**Voxel dimension**	**TR**	**TE**	**Matrix size**	**Slices**	**MR Sequence**	**TE**	**TR**	**TI**	**Matrix size**	**Slice thickness**
**London**	1.5T	General Electric Signa	3D (1.2x1.2x1.5 mm)	13.3 ms	4.2 ms	256x256	124	T2	80 ms	1720 ms	–	256x256	5 mm
	1.5T	General Electric Signa	3D (1.2x1.2x1.2)	14.3 ms	5.1 ms	256x256	156	PD-T2	17-102 ms	2000 ms		256x256	5 mm
	1.5T	General Electric Signa	3D (1.2x1.2x1.5)	29 ms	15 ms	256x256	124	PD-T2	17-102 ms	2000 ms	–	256x 256	5 mm
	3T	Philips Achieva	3D (1x1x1 mm)	6.8 ms	3.1 ms	256x256	256	PD-T2	19-85 ms	3500 ms	–	240x240	3 mm
**Milan**	1.5T	Siemen Avanto	3D (1x1x1 mm)	2000 ms	3.93 ms	256*224	208	PD-T2	28-113 ms	2560 ms	–	256x256	2.5 mm
	3T	Philips Intera	3D (0.89x0.89x1 mm)	25 ms	4.6 ms	256*256	220	PD-T2	24-120 ms	3350 ms	–	256x256	3 mm
**Graz**	3T	Siemens Tim Trio	3D (1x1x1 mm)	1900 ms	2.6 ms	176*221	256	FLAIR	69 ms	10000 ms	2500 ms	192x256	3 mm
**Barcelona**	1.5T	Siemens Symphony	3D (1x1x1 mm)	1980 ms	3.1 ms	256x256	176	FLAIR	95 ms	8500 ms	2440 ms	192x256	3 mm
	3T	Siemens Tim Trio	3D (1x1x1.2 mm)	2300 ms	2.98 ms	256x240	128	FLAIR	93 ms	9000 ms	2500 ms	400x512	3 mm
**Amsterdam**	1.5T	Siemens Vision	3D (1x1x1 mm)	4000 ms	20 ms	180x256	256	T2	20 ms	4000 ms	108 ms	256x256	3 mm
**Rome**	1.5T	Siemens Avanto	3D (1x1x1 mm)	9000 ms	89 ms	192x256	160	FLAIR	89 ms	9000 ms	2500 ms	192x256	3 mm
**Siena**	1.5T	Philips Gyroscan	2D (0.97x0.97x3mm)	35 ms	10 ms	256x256	50	FLAIR	150 ms	9000 ms	2725 ms	256x256	3mm
**PPMI**	3T	Siemens Tim Trio	3D (1x1x1mm)	2300 ms	2.52 ms	176x240	256	–	–	–	–	–	–

FLAIR, Fluid Attenuated Inversion Recovery.

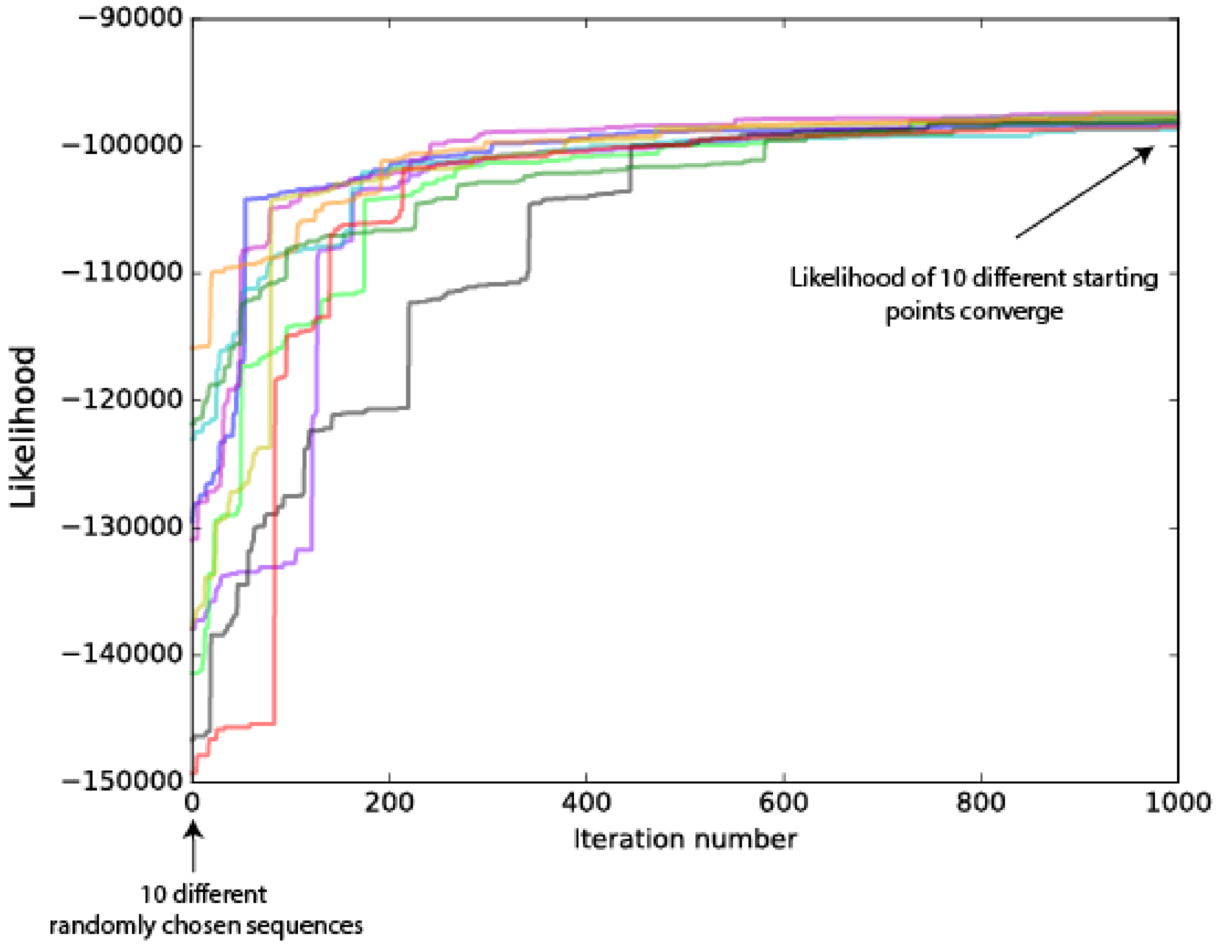

Supplementary Figure 1: Greedy ascent search and convergence of likelihoods. The most likely sequence of atrophy progression in relation to 10 randomly chosen initial sequences. The y-axis shows the data likelihood (calculated from Equation 1). The x-axis shows the number of iterations at which two events are randomly swapped in search for a higher sequence likelihood. This procedure was repeated during each cross-validation (10 times).

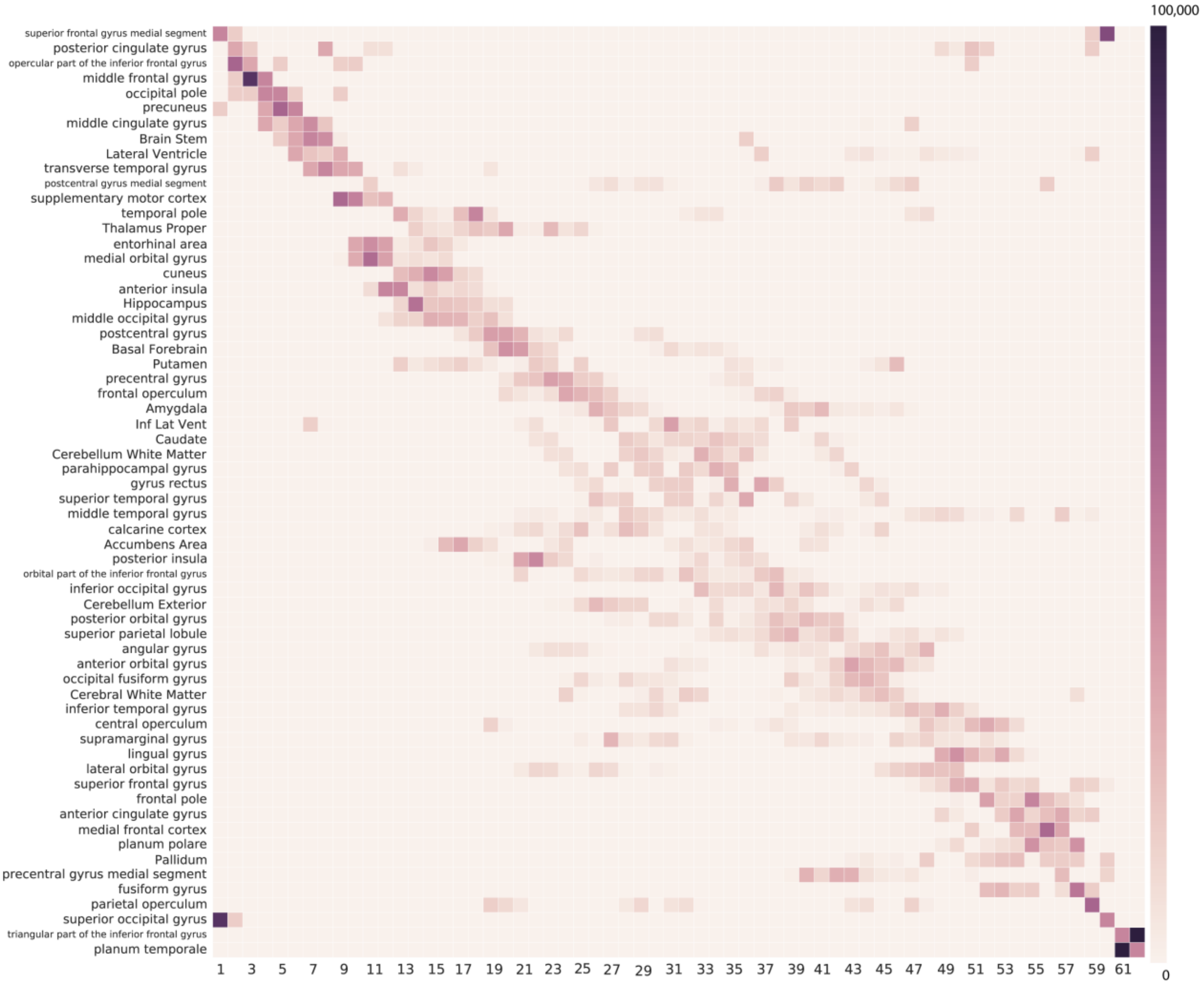

Supplementary Figure 2: Event-based model applied to all regions in patients with CIS/relapse-onset MS

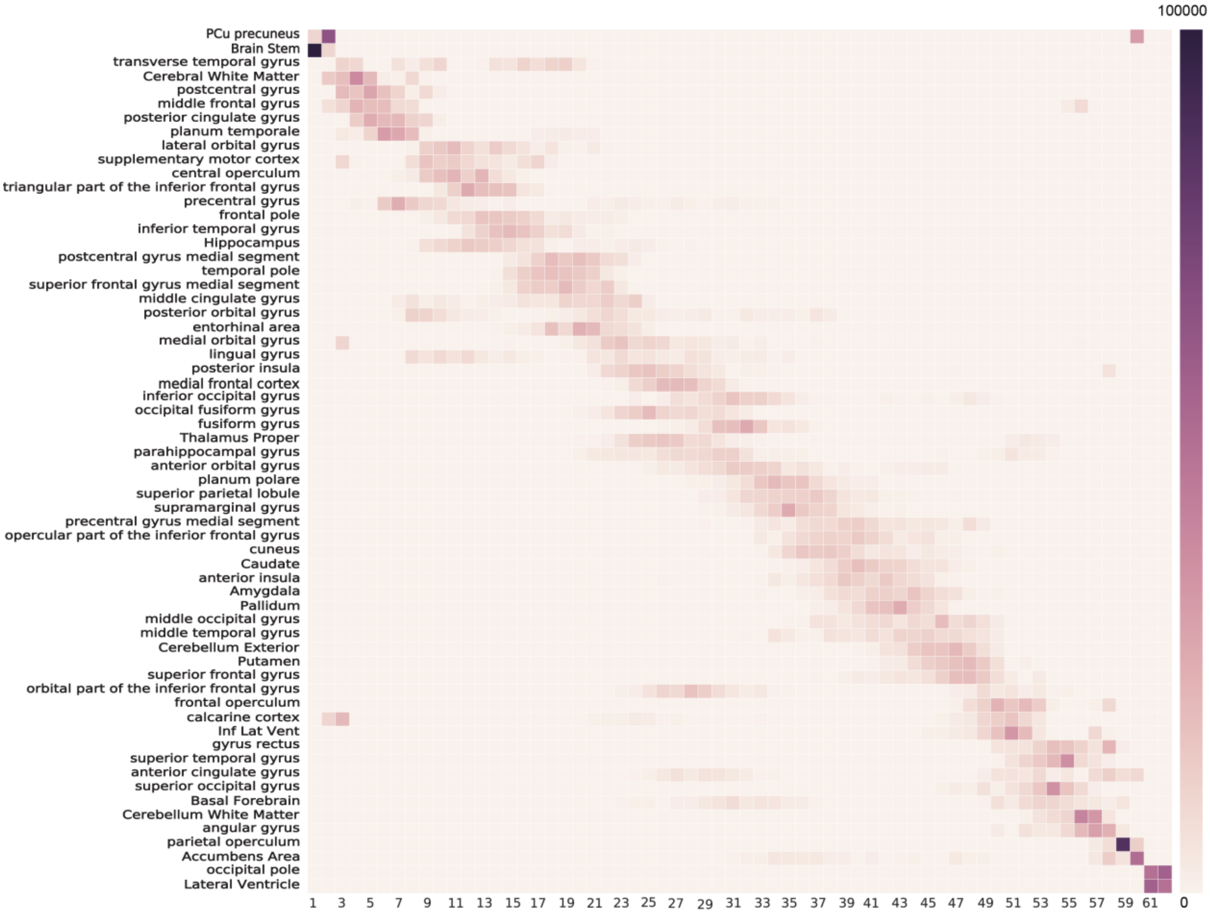

Supplementary Figure 3: Event-based model applied to all regions in patients with PPMS.
